# Complete Mitochondrial DNA Sequence of the Mucoralean Fusion Parasite *Parasitella parasitica*

**DOI:** 10.1128/genomeA.00912-14

**Published:** 2014-11-13

**Authors:** Sabrina Ellenberger, Anke Burmester, Johannes Wöstemeyer

**Affiliations:** General Microbiology and Microbe Genetics, Friedrich-Schiller-University Jena, Jena, Germany

## Abstract

The complete mitochondrial DNA sequence of the *Mucor*-related fungus *Parasitella parasitica* has been sequenced. It has a G+C content of 30% and a total length of 83,361 bp. All protein-coding genes normally found in fungi are present in the sequence. A special feature is the remarkably high number of 27 homing endonucleases.

## GENOME ANNOUNCEMENT

The mucoralean mold *Parasitella parasitica* is a facultative parasite of many zygomycetes. The parasitic lifestyle is accompanied by the fusion of specialized and spatially limited structures at the hyphal tips with the hosts (1). Parasitism is thus necessarily linked with unidirectional transport of organelles from the parasite to the host. The transfer and expression of genes residing in nuclei have been verified for antibiotic resistance markers (2) and several genes involved in amino acid biosynthesis (2, 3). In order to initiate the analysis of mitochondrial information after transfer, we sequenced the mitochondrial DNA (mtDNA) of *Parasitella parasitica*.

The *Parasitella* chondriome was obtained by Illumina sequencing (Eurofins Genomics, Ebersberg, Germany). The mitochondrial information resides on a single circular molecule with a total length of 83,361 bp. Compared with others, *P. parasitica* ranges among those fungi with larger mtDNA. For other zygomycetes, the lengths differ, with 54,178 bp in *Rhizopus oryzae* (4), 58,745 bp in *Mortierella verticillata* (4), and 62,082 bp in *Phycomyces blakesleeanus* (sequence data available at http://www.jgi.doe.gov/). The obligate parasitic species *Zancudomyces culisetae* (formerly *Smittium culisetae*) has a comparable chondriome size of 58,654 bp (4).

The *Parasitella* chondriome harbors genes for those proteins that are normally found in fungi, the small and large subunit rRNAs, and it has 26 tRNA genes. On the whole, 41 protein-coding genes were identified, some of which are split or duplicated (*atp9*, *cox1*, *cox2*, *cox3*, *cob*, *nad1*, *nad5*, *nad6*, *trnM*, and *trnV*). Both DNA strands are transcribed.

The chondriome harbors 27 genes for endonucleases (12 with LAGLIDADG domains, 8 with LAGLIDADG/HNH domains, and 7 with GYI-YIG domains). Most of them are intron situated and thus must be addressed as true homing endonucleases. mtDNAs of other zygomycetes differ in this respect. *Lichtheimia ramosa* has no genes for endonucleases (5), *R. oryzae* harbors 6, *P. blakesleeanus* 12, *M. verticillata* 6, and the considerably more distantly related parasitic harpellalean fungus *Zancudomyces culisetae* has 13 genes for homing nucleases (4).

The data support the idea that introns, rendered mobile by the acquisition of homing nuclease genes, develop an evolutionary tendency for enrichment in an organism acting as gene donor.

The mtDNA sequence of *P. parasitica* constitutes the starting point for experimental approaches that follow the fate of mitochondria after infection. Experiments along this line have become especially interesting since it is known that mitochondria are uniparentally inherited in the zygomycete *P. blakesleeanus* (6). The sequence allows the construction of parasite-specific hybridization probes to study postinfection behavior directly at the level of organelles.

### Nucleotide sequence accession number.

The mtDNA sequence is deposited in GenBank under accession no. KM382275.
